# Unveiling the Kinomes of *Leishmania infantum* and *L. braziliensis* Empowers the Discovery of New Kinase Targets and Antileishmanial Compounds

**DOI:** 10.1016/j.csbj.2019.02.005

**Published:** 2019-02-08

**Authors:** Joyce V.B. Borba, Arthur C. Silva, Pablo I.P. Ramos, Nathalia Grazzia, Danilo C. Miguel, Eugene N. Muratov, Nicholas Furnham, Carolina H. Andrade

**Affiliations:** a Labmol – Laboratory for Molecular Modeling and Drug Design, Faculdade de Farmácia, Universidade Federal de Goiás - UFG, Goiânia, GO, 74605-510, Brazil.; b Instituto Gonçalo Moniz (IGM), Fundação Oswaldo Cruz (FIOCRUZ), Salvador, BA, 40296-710, Brazil.; c LEBIL – Laboratory of Leishmania Biology Infection Studies, Department of Animal Biology, Biology Institute, State University of Campinas (UNICAMP), Campinas, SP, Brazil.; d Laboratory for Molecular Modeling, Division of Chemical Biology and Medicinal Chemistry, Eshelman School of Pharmacy, University of North Carolina, Chapel Hill, NC, 27599, USA.; e Department of Chemical Technology, Odessa National Polytechnic University, Odessa, 65000, Ukraine.; f Department of Pathogen Molecular Biology, London School of Hygiene and Tropical Medicine, London, UK.

**Keywords:** *Leishmania infantum*, *Leishmania braziliensis*, Kinome, Kinases, Drug repurposing, Target prioritization

## Abstract

Leishmaniasis is a neglected tropical disease caused by parasites of the genus *Leishmania* (NTD) endemic in 98 countries. Although some drugs are available, current treatments deal with issues such as toxicity, low efficacy, and emergence of resistance. Therefore, there is an urgent need to identify new targets for the development of new antileishmanial drugs*.* Protein kinases (PKs), which play an essential role in many biological processes, have become potential drug targets for many parasitic diseases. A refined bioinformatics pipeline was applied in order to define and compare the kinomes of L. *infantum* and L. *braziliensis,* species that cause cutaneous and visceral manifestations of leishmaniasis in the Americas, the latter being potentially fatal if untreated. Respectively, 224 and 221 PKs were identified in L. *infantum* and L. *braziliensis* overall. Almost all unclassified eukaryotic PKs were assigned to six of nine major kinase groups and, consequently, most have been classified into family and subfamily. Furthermore, revealing the kinomes for both *Leishmania* species allowed for the prioritization of potential drug targets that could be explored for discovering new drugs against leishmaniasis. Finally, we used a drug repurposing approach and prioritized seven approved drugs and investigational compounds to be experimentally tested against *Leishmania*. Trametinib and NMS-1286937 inhibited the growth of L. *infantum* and L. *braziliensis* promastigotes and amastigotes and therefore might be good candidates for the drug repurposing pipeline.

## Graphical Abstract

**Figure:**
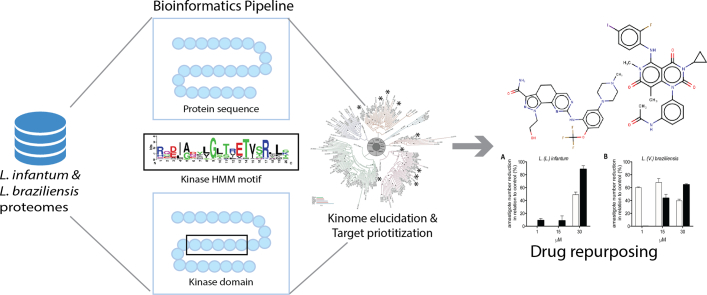

## 1 Introduction

Leishmaniasis is a parasitic disease caused by the etiologic agent *Leishmania* spp. The parasites are transmitted to humans through the bite of infected phlebotomine sandflies from the *Lutzomyia* and *Phlebotomus* genera. [1]. The disease is clinically classified based on its manifestations as Visceral Leishmaniasis (VL) and Cutaneous Leishmaniasis (CL) and on the *Leishmania* species parasitizing the host. Two important human pathogen species are *Leishmania infantum,* which cause New World and Old World VL, and *Leishmania braziliensis,* which is among the species causing CL in the Americas [[2], [3], [4]].

The countries most affected by leishmaniasis are in Africa, Asia, and Latin America. It is estimated that about 0.2 to 0.4 million new cases of VL and 0.7 to 1.2 million new cases of CL appear each year. Yearly, there are around 20,000–40,000 deaths in the world related to the disease [5,6]. The current treatment of VL and CL rely on pentavalent antimonials - amphotericin B, paromicine, pentamidine, and miltefosine - which have issues with toxicity and administration. In addition, their effectiveness is compromised due to the emergence of resistant strains. Hence, there is a need for developing new drugs against leishmaniasis [7,8].

Protein kinases are among the largest protein families coded in the genome of most organisms, constituting ~2% of the diversity of eukaryotic genomes [9]. They are mediators of many regulatory, signal transduction, and cell development pathways [10]. Thus, a considerable research effort to select molecular targets for new compounds is centered around protein kinases [[11], [12], [13]]. Protein kinases exercise their role by phosphorylating other molecules [13]. Eukaryotic kinases (ePK) have a very conserved domain composed of 11 subdomains and their tridimensional structure has a *N*-terminal lobe with an antiparallel β-sheet and a *C*-terminal lobe with α-helices [14]. Proteins that transfer phosphates from ATP to other biomolecules and do not have the eukaryotic kinase domain are termed atypical protein kinases (aPK) and protein kinase like (PKL) [15].

ePKs are classified according to the amino acid they phosphorylate: serine/threonine protein kinases or tyrosine protein kinases [16]. They are further classified into 9 groups, based on their sequence similarity, according to the Manning classification [14]: (i) the AGC group – protein kinases A, G, and C; (ii) CAMK group – Ca^+^/CAM-dependent kinases; (iii) CMGC group – CDK, MAPK, GSK3, and CLK; (iv) CK1 – casein kinase 1; (v) STE group – homologs of yeast sterile 7, 11, and 20; (vi) RGC group – receptor guanylate cyclases (vii) TK group – tyrosine kinase; (viii) TKL group – tyrosine kinase-like; and (ix) “Other” group – several kinase families that do not fit within any of the other main kinase groups [[15], [16], [17]].

**Fig. 1:**
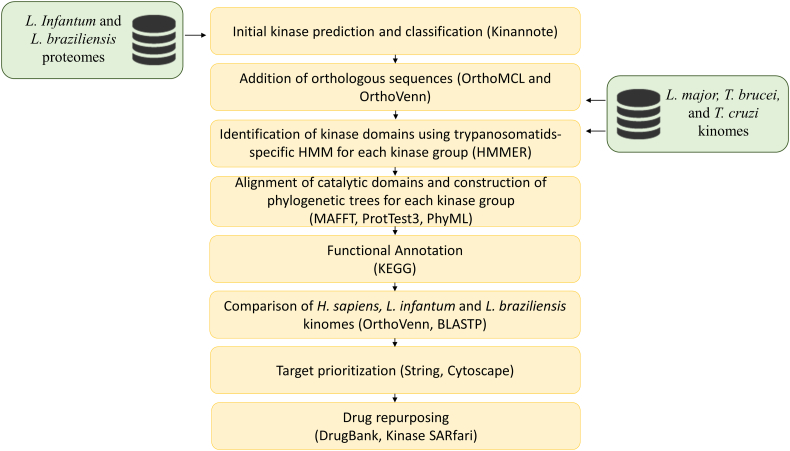
Bioinformatics pipeline used to define and characterize *L. infantum* and *L. braziliensis* kinomes, prioritize kinase targets and select drugs to target *Leishmania* protein kinases*.*

Given that (a) protein kinases have essential roles in the cell [10]; many human kinase inhibitors have been successfully applied especially in cancer therapy [18]; and that (b) kinases have conserved structures and functions [12], revealing the L. *infantum* and L. *brazilienis* kinomes may accelerate the drug discovery process for leishmaniasis. Here, we have elucidated for the first time the kinomes of L. *infantum* and L. *braziliensis*, species that cause visceral and cutaneous leishmaniasis, respectively, in the Americas. We developed and applied a robust bioinformatics pipeline that enabled us to classify most of the protein kinases at a subfamily level. We also applied a drug repurposing workflow that prioritized novel protein kinases that are essential for the parasite's survival or are central in a protein interaction network. Moreover, we selected and experimentally evaluated some kinase inhibitors that might inhibit some of these targets. The general workflow of this study is presented in Fig. 1.

## 2 Material and Methods

### 2.1 Definition and Prediction of L. *infantum* and *L. braziliensis* Kinomes

We performed a proteome-wide analysis of PKs of the species L. *infantum* and *L. braziliensis using a mod*ified and refined bioinformatics pipeline described elsewhere [19]. Briefly, the proteomes of L. *infantum* and *L. braziliensis* were inputted into the program Kinannote v.1.0 [20]. The kinases were classified into groups, families, and, ultimately, subfamilies. Proteins with partial classification or that were unclassified were kept for further manual curation. The *L. major* kinome [21] was used as a reference to further classify the unclassified and partially classified kinases, to improve their classification, and to find proteins that were not detected by Kinannote. In order to precisely compare *L. major, L. infantum,* and *L. braziliensis* kinomes, we predicted the orthologous sequences from the proteomes of the 3 species using the program OrthoMcl v.2.0.9 [22]. InterproScan v.5.18 (https://www.ebi.ac.uk/interpro/search/sequence-search) was used to elucidate and localize kinase domains of the classified proteins. We also constructed HMM profiles for individual kinase groups based on closely related organisms' kinase classifications, then searched these profiles through the proteomes of L. *infantum* and *L. braziliensis*. The HMM profile construction and search was done based on the catalytic domain sequence of individual kinase groups of *L. major*, *T. brucei*, and *T. cruzi* protein kinases using HMMer v. 3.1b2 (http://hmmer.org/) software.

### 2.2 Phylogenetic Tree Construction

In order to study the relationships within the *L. infantum* kinases from each group, multiple phylogenetic trees were constructed. For each group, only the catalytic domains were kept for automatic multiple sequence alignment (MSA) using MAFFT v. 7.215 [23] in most accurate mode (L-INS-i; parameters *–localpair –maxiterate 1000*). Next, the alignments were improved using the *–refine* switch in MUSCLE v. 3.8.31 [24]. Biopython scripts [25] were used to convert between the MSA formats generated by the distinct tools. ProtTest3 v. 3.4.2 was used to select the best-fit model of amino acid replacement according to the Akaike information criterion measure [26]. PhyML v. 20,131,022 [27] was used to infer maximum likelihood trees with 1000 bootstrap replicates using the amino acid substitution model chosen in the previous step. FigTree v. 1.4.3 (available at http://tree.bio.ed.ac.uk/software/figtree/) was used to perform tree visualization, editing, and export.

### 2.3 Functional Annotation

The final list of classified protein kinases was functionally annotated by searching the KEGG BRITE (http://www.kegg.jp/blastkoala/) and Gene Ontology (http://www.geneontology.org/) databases; a consensus classification was manually annotated.

### 2.4 Comparison of *L. infantum, L. braziliensis* and *Homo sapiens* Kinomes

The comparison of the 3 kinomes was done using the software OrthoVenn (http://probes.pw.usda.gov/OrthoVenn/) to infer which proteins cluster together and to find kinases present in both *Leishmania* species and absent in humans. Pairwise alignment of the kinomes was performed using the BLASTP algorithm v. 2.2.24 [28] locally at default parameters.

### 2.5 Drug Target Prediction and Prioritization

In order to select potential drug targets among the kinomes, we performed an essentiality search by selecting *L. infantum* proteins homologous (BLASTP; e-value ≤10^−30^) to *T. brucei* kinases with lethal siRNA phenotypes – found at Tritrypdb (http://tritrypdb.org/tritrypdb/). A complementary target prioritization approach was performed by constructing a protein network interaction of kinase proteins through STRING [29] v. 10.0 (https://string-db.org/) web server and the resulting network was analyzed from a graph-theoretic perspective. Topological measures of centrality in this network were calculated using the CytoNCA Cytoscape v.3.3.0 plugin [30].

### 2.6 Compound Selection for Experimental Evaluation

The FASTA sequences of each prioritized target were used to interrogate two different publicly available databases that provide detailed information on drugs and their targets: DrugBank [31] and kinase SARfari (https://www.ebi.ac.uk/chembl/sarfari/kinasesarfari/). The search strategy was based on the principle of homology, where each query (*L. infantum* targets) was compared for matches to known drug targets contained in each database. We set a strict threshold on the E-value ≤10^−30^ to consider the target as acceptable and we only considered approved drugs or compounds in clinical trials for this search. We also searched for “druggability” of the targets in kinase SARfari. Then, a list of drugs/compounds and their possible targets was compiled. A literature search was carried out using the PubMed and PubChem databases and SciFinder engines to identify which of the compounds related to the selected targets have not been evaluated against *Leishmania* species. The details of the search were in the format: (“drug name” [MeSH Terms] OR “drug name” [All Fields]) AND (“*Leishmania”* [MeSH Terms] OR “Leishmania” [All Fields]). After applying the literature search filter, the antileishmanial activity of these compounds were predicted using an in-house QSAR phenotypic model for L. *infantum.* The compounds predicted to be active by the QSAR model were purchased for *in vitro* experimental evaluation.

### 2.7 *In vitro* Evaluation of Selected Compounds in *Leishmania spp*

Fresh aliquots from 10 mM DMSO (Sigma-Aldrich)-diluted stock solutions of seven kinase inhibitors (Selumetinib, Refametinib, MEK162, MLN8054, RG1530, NMS-1286937 and Trametinib; purchased from MedChemExpress) were prepared and tested against *L. (L.) amazonensis* (MHOM/BR/PH8), *L. (L.) infantum* (MHOM/BR/1972/LD) and *L. (V.) braziliensis* (MHOM/BR/94/H3227) promastigotes as described elsewhere [32]. Compounds with good antileishmanial activity were tested against *L. (L.) infantum* and *L. (V.) braziliensis* intracellular amastigotes.

Parasites were maintained at 26 °C in medium 199 (Sigma-Aldrich) as previously described [33]. To perform the promastigote assays, approximately 5 × 10^6^ logarithmic-phase promastigotes were incubated with increasing concentrations of each kinase inhibitor (1, 5, 10, 20, 75, and 100 μM) in triplicates. Viability was assessed by the MTT method and 50% of effective concentrations (EC_50_%) were determined by sigmoid regression analysis using Prism 5.0a (GraphPad Software. Inc.).

To perform the amastigote assays, 4 × 10^5^ BALB/c bone marrow derived macrophages (BMDM) were obtained [34] and infected with *L. (L.) infantum* and *L. (V.) braziliensis* stationary-phase promastigotes (at a ratio of 10 parasites; 1 BMDM) for 24 h in 24 well plates and kept at 37 °C and 34 °C, respectively. The protocol was approved by the Animal Experimentation Ethics Committee (CEUA-UNICAMP #4535-1/2017). Established infections were incubated with 10, 15 and 30 μM of NMS-1286937 and Trametinib. Parasite burden was assessed by counting intracellular amastigotes in at least 200 BMDM per coverslip. Each assay was performed in triplicates and the reduction in amastigote number was compared to the untreated infection group (100%).

## 3 Results

### 3.1 Definition and Prediction of *L. infantum* and *L. braziliensis* Kinomes

#### 3.1.1 *L. infantum* Kinome

We developed and applied an integrative bioinformatics pipeline (Fig. 1) that allowed us to identify 197 protein kinases in the *L. infantum* proteome. From these original 197, 40 kinases were assigned to subfamilies, 109 to families, 18 to groups, and 30 remained unclassified. After manual curation and improvement of this draft kinome (see Methods Section), we obtained a total of 224 kinases (196 ePKs, 28 PKLs/aPKs), of which 157 were assigned to subfamilies, 64 to families, three to groups, and only one remained unclassified (Table 1).

**Table 1:** *Leishmania infantum* and *L. braziliensis* kinome classification before and after curation. The classification into families and subfamilies was improved and most kinases that were not classified in the initial steps could be further identified by manual curation.

	Group	Family	Subfamily	Unclassified
*L.infantum* (Draft)	18	109	40	30
*L. infantum* (Final)	2	64	157	1
*L. braziliensis* (Draft)	22	114	31	28
*L.braziliensis* (Final)	3	61	155	2

A total of 195 ePKs were identified in L. *infantum* proteome. There were representatives of six kinase groups (Table 2), among which 12 kinases were classified into the following families of AGC group: protein kinase B (AKT, *n* = 1); nuclear DBF2-related kinases (NDR, n = 1); phosphoinositide dependent protein kinase 1 (PDK1, *n* = 3); protein kinase A (PKA, n = 3); and ribosomal S6 kinase (RSK, *n* = 2). Two AGC kinases were not assigned to any family.

**Table 2:** Kinase's group and family classification after curation. The number of proteins in each group increased after manual curation and almost all were assigned to families; only a few unclassified proteins remained in the end of the curation pipeline. The most representative groups were CMGC, STE, and “Other” and the least representative groups were AGC, CAMK, and CK1.

Group	Family	*L. infantum*	*L. braziliensis*
AGC	AKT	1	1
	NDR	1	1
	PDK1	3	3
	PKA	3	2
	RSK	2	1
	NA⁎	2	2
	Total	12	10
CAMK	CAMK1	6	5
	CAMKL	11	11
	CAMK_Unique	4	5
	CDPK	2	2
	Total	23	23
CMGC	CDK	14	12
	CK2	2	2
	CDKL	0	2
	CLK	4	4
	DYRK	9	9
	GSK	2	2
	MAPK	13	13
	RCK	3	3
	SRPK	3	2
	Total	50	49
CK1	CK1	6	6
	TTBK	1	1
	Total	7	7
STE	STE11	31	31
	STE20	2	2
	STE7	7	7
	STE-Unique	1	1
	Total	41	41
Other	AUR	3	3
	Bud 32	1	1
	CAMKK	4	4
	IKS	1	1
	NAK	2	2
	NEK	24	23
	PEK	3	3
	PLK	1	1
	SCY1	2	2
	TLK	1	1
	ULK	3	3
	VPS15	1	1
	WEE	2	1
	Other-unique	14	14
	NA⁎	0	1
	Total	62	61
Unclassified		1	2
Total epk		196	193
Atypical	A6	1	1
	PDHK	3	3
	Total	4	4
PKL	PIK	5	4
	PIKK	6	6
	RIO	2	2
	ABC1	5	5
	alpha	4	5
	CAK	2	2
	Total	24	24
Total PK		224	221

⁎ NA = Not assigned to a family.

**Fig. 2:**
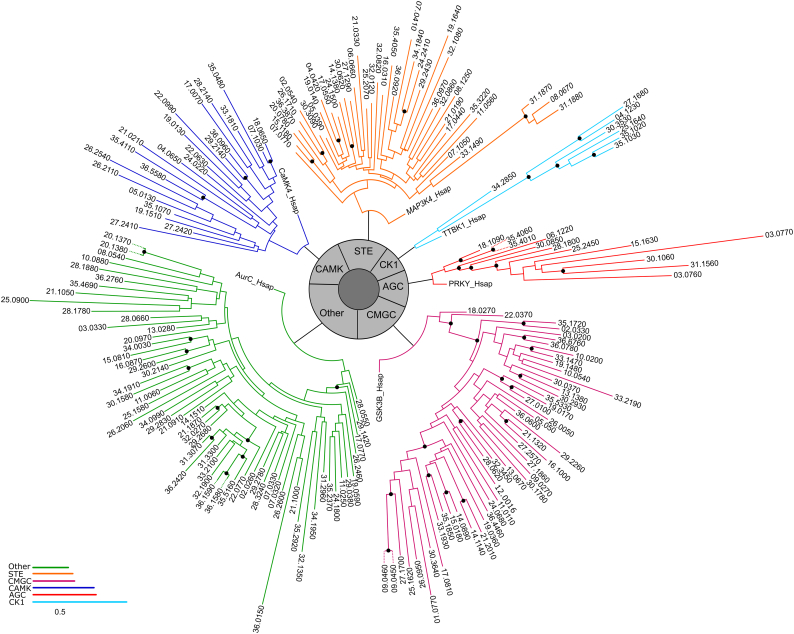
Phylogenetic analysis of the ePK groups of *L. infantum.* The catalytic domains of *L. infantum* ePKs were used to construct ML trees using PhyML program. Each of the six kinase groups is highlighted in a different color and black circles indicate bootstrap support values (1000 replicates) equal or higher than 60%.

Twenty-three kinases were classified into the following families of CAMK group: CAMK family 1 (CAMK1, n = 6); calcium/calmodulin protein kinase-like (CAMKL, n = 11); CAMK-Unique (*n* = 4); and the calcium-dependent protein kinase (CDPK, *n* = 2). The group CK1 had 7 members, of which 6 were identified as cell kinase 1 family (CK1) and one was from the tau tubulin kinase family (TTBK).The CMGC group is one of the most represented in *L. infantum* kinome, with 50 protein kinases classified in the following families: cyclin-dependent protein kinase (CDK, *n* = 14); cell kinase 2 (CK2, *n* = 2); CDC-like kinase (CLK, *n* = 4) dual-specificity tyrosine-regulated kinase (DYRK, *n* = 9); glycogen synthase kinase 3 (GSK, n = 2); mitogen-activated protein kinase (MAPK, *n* = 13); resistance to complement killing (RCK, *n* = 3); and SR protein kinase (SRPK, n = 3). Forty-one PKs were assigned to the following families of STE group: MAP kinase kinase kinase genes, homologous to yeast Ste. 11 (STE11, *n* = 31); MAP kinase kinase kinase kinase genes, homologous to yeast Ste20 (STE20, *n* = 2); MAP kinase kinase genes, homologous to yeast Ste. 7 (STE7, *n* = 7); and STE-Unique (*n* = 1). The group “Other” contains several kinase families that do not fit within any of the main kinase groups. There were 62 kinases classified into the families of this group: aurora kinase family (AUR, *n* = 3); Bud 32 (n = 1); calcium/calmodulin-regulated kinase kinase (CAMKK, *n* = 4); IRA 1 kinase suppressor (IKS, n = 1); numb-associated kinase (NAK, *n* = 2); mitotic kinase (NEK, *n* = 24); PEK (n = 3); polo-like kinase (PLK, n = 1); SCY1 (n = 2); tousled-like kinase (TLK, n = 1); Unc51-like kinase (ULK, n = 3); VPS15 (n = 1); WEE (n = 2); and Other-unique (*n* = 14). Specific information on each classified kinase can be found in Supplementary Table S1 online. Moreover, a phylogenetic tree of the L. *infantum* ePK kinome groups (Fig. 2) was constructed and representative human kinases of each group were used to root each tree. The tree was consistent with our classifications and comparative analysis.

Besides ePKs, 24 protein kinase like (PKL) proteins were identified, and were distributed into phosphatidyl-inositol kinase (PIK, *n* = 5); phosphatidyl-inositol3 kinase related kinases (PIKK, *n* = 6); right open reading frame kinase (RIO, *n* = 2); ABC1 (n = 5); alpha (*n* = 4); and CAK (n = 2). We identified four atypical protein kinases, of which one was classified into the A6 family and three were classified into the pyruvate dehydrogenase kinase family (PDHK).

We also assessed the transcription profile of L. *infantum* kinases in promastigotes. We searched for RNA-seq evidence of the species transcriptome in TriTrypDB [35]. A histogram plot was constructed using the percentile of expression of each kinome protein in *Leishmania* promastigotes (see Supplementary Fig. S1). There are 33 highly expressed kinases in L. *infantum* which rank at the upper quartile of expression. At the lower quartile of expression there are 84 kinases. The other 139 kinases are constitutionally expressed. Most of the highly expressed kinases are members of the CMGC group, probably due to the diverse and important functions of MAPK cascades and CDKs [36].

#### 3.1.2 *L. braziliensis* Kinome

The draft kinome of *L. braziliensis* contained 195 kinases, 31 of which could be classified on the level of subfamilies; 114 on the level of families; 22 on the level of groups; and 28 protein kinases remained unclassified. After manual curation (see Methods), 221 proteins (193 ePKs and 28 PKL/ aPKs) were defined as protein kinases and classified into groups (*n* = 3); families (*n* = 61); subfamilies (*n* = 155); and 2 protein kinases remained unclassified (Table 1).

A total of 193 ePKs were identified in the L. *braziliensis* proteome, among which there were representatives of 6 kinase groups (Table 2). The AGC group contained 10 kinases classified in the following families: AKT (*n* = 1); NDR (n = 1); PDK1 (*n* = 3); PKA (*n* = 2); and RSK (n = 1). Two AGC kinases were not assigned to any family. The CAMK group had 23 kinases classified in the following families: CAMK1 (*n* = 5); CAMKL (*n* = 11); CDPK (*n* = 2); and CAMK-Unique (n = 5). The CK1 group had 7 kinases classified into CK1 (n = 6) and TTBK (*n* = 1) families. There were 49 proteins assigned to the following families of CMGC group: CDK (*n* = 12); CK2 (n = 2); CDKL (n = 2); CLK (*n* = 4); DYRK (*n* = 9); GSK (n = 2); MAPK (*n* = 13); RCK (*n* = 3); and SRPK (*n* = 2). Among the STE group, there were 41 kinases classified in the following families: STE11 (*n* = 31); STE20 (n = 2); STE7 (*n* = 7); and STE-Unique (n = 1). The “Other” group contained 60 kinases within the following families: AUR (n = 3); Bud32 (n = 1); CAMKK (*n* = 4); IKS (n = 1); NAK (n = 2); NEK (*n* = 23); PEK (n = 3); PLK (n = 1); SCY1 (n = 2); TLK (n = 1); ULK (n = 3); VPS15 (n = 1); WEE (n = 1); and Other-Unique (*n* = 14). One “Other” kinase was not assigned to any family.

Along with the ePKs, there were 24 PKLs identified in L. *braziliensis* proteome. They were classified in the following families: ABC1 (*n* = 5); alpha (n = 5); PIK (n = 4); PIKK (*n* = 6); RIO (n = 2); and CAK (n = 2). There were also four atypical protein kinases: three PDHKs and one A6. Since the kinomes L. *infantum* and L. *braziliensis* are very similar, their phylogenetic trees are very similar as well and therefore we decided not to report the tree for L. *braziliensis.* Specific information of each classified kinase can be found at Supplementary Table S2.

### 3.2 Comparison between *L. infantum, L. braziliensis,* and *Homo sapiens* Kinomes

**Fig. 3:**
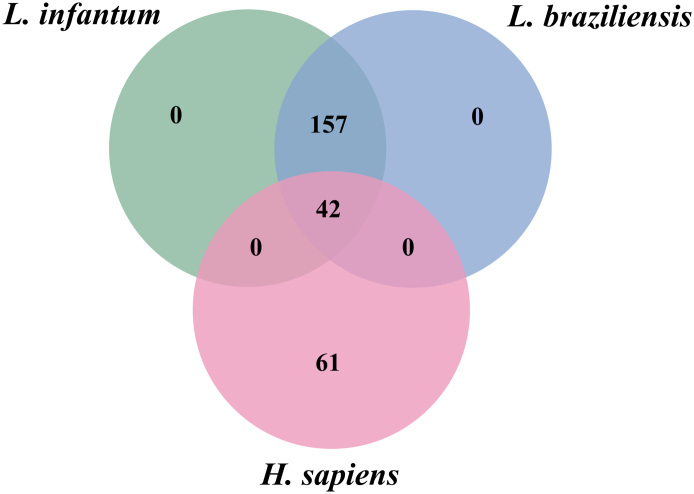
Venn diagram comparing the clusters of orthologous kinases in *L. infantum, L. braziliensis,* and *H. sapiens.* The PKs were grouped in clusters of orthologous sequences and the groups that shared proteins of the different organisms were partitioned according to the diagram.

We compared the kinomes of both *Leishmania* species and the human kinome in order to identify candidate drug targets among the parasites kinome (Fig. 3). The rationale of this approach is that kinases of both *Leishmania* species that cluster together and that do not cluster with human kinases may be considered plausible targets. If a compound inhibits one of these proteins, there is a chance that it can lead to an improvement in CL and VL while having less side effects due to off-target effects. OrthoVenn was used to infer the orthologous clusters of kinases using the sequences of the proteins from *L. infantum*, *L. braziliensis*, and *H. sapiens* kinomes. There were 42 clusters of common orthologous sequences for all three species and 157 clusters that had only *L. infantum* and *L. braziliensis* orthologous sequences. These 157 clusters were considered for further analysis for target prioritization, as they show the least similarity to human kinases.

We conducted a pairwise analysis of the *L. infantum* and *L. braziliensis* kinases with the orthologous kinases of *L. major* aiming to compare the similarity between these species kinomes. The *L. major* kinome has already been elucidated [21] and our goal was to understand how similar the orthologous kinases are to both *Leishmania* kinomes, since *L. major* was used as a reference in one of the curation steps. The overall range of identity between *L. infantum* and *L. major* proteins was between 83% to 100%, with greater diversity shown between L. *braziliensis* and *L. major* proteins, with a range of identity between 38% to 99%*.* Kinase groups of both L. *infantum* and L. *braziliensis* demonstrated high similarity to *L. major* groups, indicating that the *L. major* kinome was a good reference to infer these organisms' kinomes. We compared L. *infantum* kinome with L. *braziliensis* to know if it is possible to extrapolate our target prioritization approach. The overall range of identity between *L. infantum* and *L. braziliensis* proteins was between 29 and 99%. When comparing individual groups, those with higher shared identities were AGC, CK1, and CMGC; the “Other” group has the largest range in identity (29–96%). Likewise, we performed a pairwise comparison of *Leishmania* kinases and *H. sapiens* homologs [14] that revealed a lower range of identity: from 23 to 69% (*L. infantum* v *H. sapiens*), and from 21 to 69% (*L. braziliensis* v *H. sapiens*). This demonstrates that *Leishmania* kinases can be targeted by compounds that are unlikely to bind human kinases (Table 3 and Supplementary Table S3).

**Table 3:** Pairwise comparisons of L. *infantum* kinase sequences with orthologues in L. *braziliensis*, *L. major,* and human. BLAST analyses were performed for each sequence in each group. The table shows the range of identity in the kinase groups between the L. *infantum* proteins and the organism's proteins listed in each column.

Groups	*L. infantum x L. braziliensis* identity % range (mean ± SD)	*L. infantum x L. major* identity % range (mean ± SD)	*L. infantum x H. sapiens* identity % range (mean ± SD)	L. *braziliensis* x *L. major* identity % range (mean ± SD)	*L. braziliensis x H. sapiens* identity % range (mean ± SD)
AGC	53–96 (81.2 ± 14.9)	82–99 (93.4 ± 5.45)	25–53 (41.58 ± 8.06)	50–93 (78 ± 14.43)	25–53 (37.2 ± 7.74)
CAMK	62–97 (82.7 ± 10.11)	88–100 (94.82 ± 3.34)	23–53 (34.25 ± 7.23)	63–99 (84.39 ± 10.18)	27–69 (41.48 ± 11.19)
CK1	63–98 (80.6 ± 11.02)	90–99 (94.3 ± 3.5)	31–69 (47.7 ± 13.19)	55–95 (72.14 ± 13.88)	23–37 (31.14 ± 4.95)
CMGC	56–99 (83.6 ± 11.0)	84–99 (94.4 ± 4.1)	25–59 (40.66 ± 9.03)	59–97 (82.06 ± 10.04)	28–55 (39.1 ± 7.58)
STE	44–96 (75.5 ± 11.9)	56–99 (90.7 ± 7.69)	23–48 (34.02 ± 5.18)	54–96 (74.95 ± 10.04)	24–48 (34.6 ± 5.76)
Other	29–96 (74.3 ± 14.7)	83–99 (92.38 ± 5.19)	24–67 (34.02 ± 8.16)	38–97 (76.93 ± 12.68)	21–62 (35.29 ± 7.65)

**Fig. 4:**
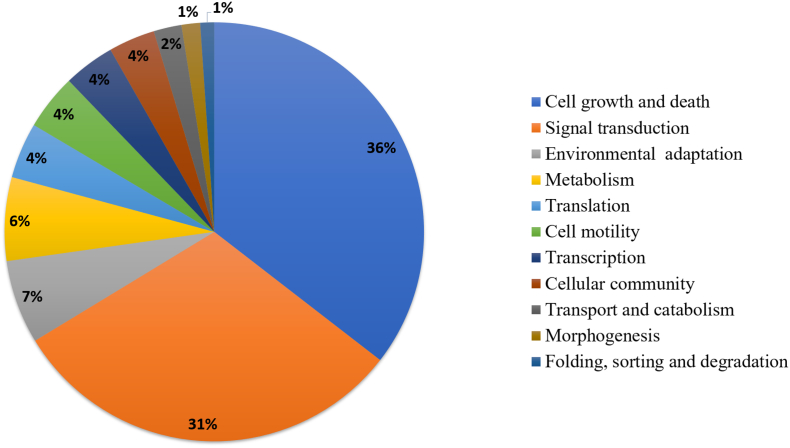
Functional annotation of *L. infantum and L. braziliensis* protein kinase. Protein kinases were distributed into pathways considering KEGG's second level of functional classification.

The 157 *Leishmania-*specific protein kinases were then functionally annotated into 3 levels of KEGG pathways and a pie chart considering the 2nd level was constructed with 11 functional categories (Fig. 4 and Supplementary Table S3 online). The most represented functions of kinome proteins were (i) cell growth and death (36%); (ii) signal transduction (31%); (iii) environmental adaptation (7%); and (iv) metabolism (6%).

### 3.3 Drug Target Prediction and Repurposing Pipeline

Two bioinformatics approaches were used for target prediction: (i)protein essentiality and (ii) centrality measures of the kinome protein-protein interaction graph. For the first approach (i), we used RNAi validations of targets with lethal phenotype in closely related *T. brucei* to infer the L. *infantum* orthologous that are essential to survival of the parasite. In the second approach, we constructed a protein interaction network using the kinome proteins as input to the STRING web server, then using the results in Cytoscape to calculate two network metrics: closeness and betweenness centralities. These may be considered as proxies for node importance in the interaction network based on their connections to other proteins. Further, the selected proteins were used to build a sub-network and the measure of in- and out-degrees was calculated to select the top connected proteins into the sub-network (Supplementary Fig. S2). Combining these complementary approaches led to the prediction of 30 potential new drug targets (Supplementary Table S4).

**Fig. 5:**
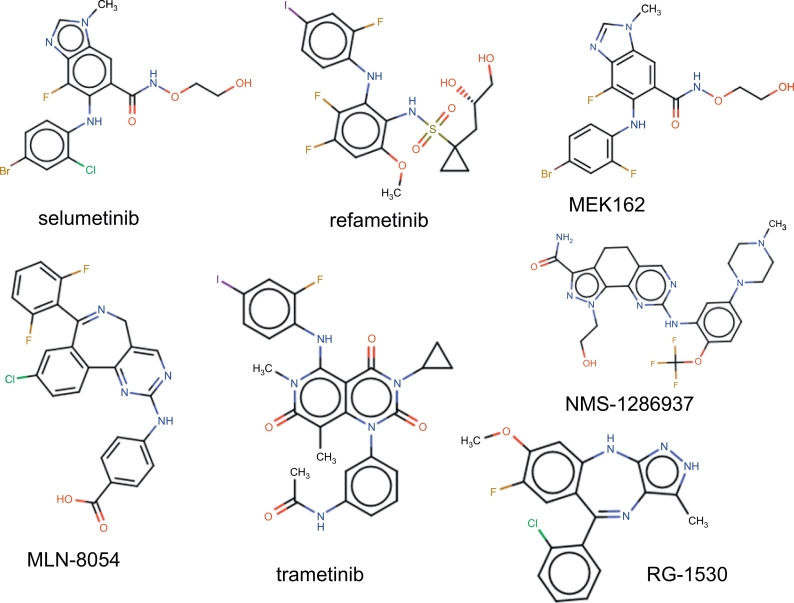
Approved drugs and investigational compounds in clinical trials prioritized in the drug repurposing pipeline and selected for experimental evaluation.

Finally, the FASTA sequences of the 30 prioritized targets were used to interrogate the publicly available databases DrugBank and Kinase SARfari (Supplementary Fig. S3) in a drug repurposing pipeline. In this step, the *E*-value threshold of ≤10^−30^ was adopted to provide high confidence for the data. This analysis predicted 11 targets associated with 42 drugs (see Supplementary Table S5). These drugs can possibly also target *Leishmania* since the sequence conservation between the human and *Leishmania* targets is high. Then, we conducted a literature search of the 42 drugs, in order to check which of them have not been tested in *Leishmania* yet. We found that 15 of those drugs have already been tested in *Leishmania*, some of them had IC_50_ lower than 10 μM [37], demonstrating that our approaches of target selection and drug repurposing were successful. The 27 compounds that have not been tested in *Leishmania* were submitted to an in-house developed QSAR model for prediction of phenotypic activity against *L. infantum*. Seven of them were predicted to be active against *L. infantum* amastigotes (Fig. 5). These compounds were then purchased and experimentally evaluated against *L. infantum, L. amazonensis,* and *L. braziliensis* promastigotes and amastigotes.

### 3.4 Experimental Validation

The kinase inhibitors selumetinib, refametinib, MEK162, MLN-8054, trametinib, NMS-1286937 and RG-1530 were selected and tested against L. *infantum,* L. *braziliensis* and *L. amazonensis* promastigotes. Selumetinib, refametinib, MEK162, MLN-8054 inhibited ~ 70% promastigote growth at concentrations higher than 75 μM, showing poor leishmanicidal activity. On the other hand, R1530, NMS-1286937 and Trametinib presented good to moderate antileishmanial activity against *Leishmania* spp. (Table 4).

**Table 4:** R1530, NMS-1286937 and Trametinib activity against *Leishmania* spp. promastigotes.

	Kinase inhibitor	EC_50_% (μM) [95% CI]
*L. (L.) amazonensis*	RG-1530	61.5 [52.5–70.5]
	NMS-1286937	29.9 [26.2–33.6]
	Trametinib	23.1 [19.8–26.4]
*L. (L.) infantum*	RG-1530	77.5 [69.7–85.3]
	NMS-1286937	13.2 [10.0–16.5]
	Trametinib	63.6 [57.8–68.8]
*L. (V.) braziliensis*	RG-1530	62.4 [55.7–69.1]
	NMS-1286937	37.3 [31.8–42.8]
	Trametinib	68.3 [64.2–72.4]

**Fig. 6:**
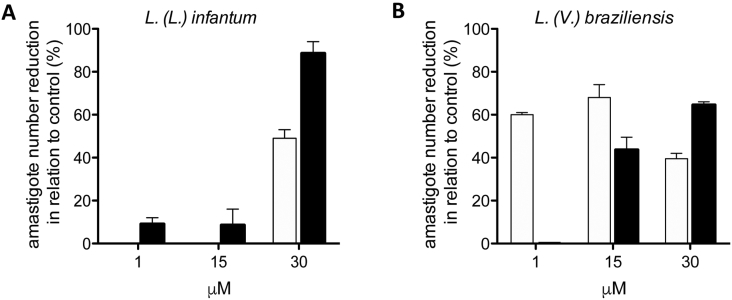
Percentage of L. *infantum* (A) and L. *braziliensis* (B) intracellular amastigote reduction after incubation with NMS-1286937 (white bars) and Trametinib (black bars) in relation to control untreated infections (100%).

Cytotoxicity assays showed that RG-1530 was toxic to bone marrow derived macrophages (BMDM) from BALB/c mice while NMS-1286937 and Trametinib led to ~15% viability reduction at 50 μM (Supplementary Fig. S4 online). For this reason, the antileishmanial activity of NMS-1286937 and Trametinib were evaluated against intracellular amastigotes. NMS-1286937 at 30 μM reduced approximately 50% of *L. (L.) infantum* parasite burden whilst Trametinib showed a more pronounced effect at the same dose (~90% reduction) (Fig. 6). For *L. (V.) braziliensis*, both compounds reduced ~50% of intracellular amastigotes at 15 and 30 μM (Fig. 6).

## 4 Discussion

Our bioinformatics pipeline, expanding on established methods for kinase identification, enabled us to complete the first complete definition and classification of the kinomes of two *Leishmania* species important to human health. The draft kinome revealed 197 and 195 protein kinases in *L. infantum* and *L. braziliensis*, respectively. These numbers represent 2.35% and 2.08% of these species' genomes. The curation procedure further improved that number to 224 and 221, representing 2.67% and 2.35% of their genomes. The number of protein kinases is compatible with related trypanosomatid kinomes, which comprise approximately 2% of their genomes [38] and is also comparable to other parasites, such as *Plasmodium falciparum* (1.5%) [39] and *Schistosoma mansoni* (1.9%) [36]. Organisms from the KinBase kinome database (www.kinase.com), which does not contain trypanosomatid kinomes, have a similar number of kinases compared with their genomes ranging from 1.5 to 2% [36].

The large set of PKs in the genus suggests that phosphorylation is a very important process in parasite biology. There are representatives of six of the nine ePK groups are described by Manning et al. [14]. Tyrosine Kinase (TK), Tyrosine Kinase Like (TKL), and Receptor Guanylate Cyclase (RGC) groups are not represented. The phosphorylation of tyrosine residues has been reported in trypanosomatids [[40], [41], [42], [43]]. This phenomenon was related to the action of atypical tyrosine kinases such as Wee1 [44] and dual-specific protein kinases such as DYRK, CLK, and STE7. CRK3 may also be involved in tyrosine phosphorylation since it has a tyrosine residue in a regulatory subdomain [45].

In comparison with the previously elucidated *L. major kinome*, the kinomes of *L. infantum* and *L. braziliensis* constituted a larger number of proteins (total of 199 protein kinases in L. major kinome) [38]. This difference may be due to intra-species variability or the more detailed approach used in the current work. Also, the authors of the L. *major* kinome study [21] could only assign the kinases into groups and families; our investigation led to a more detailed classification and could assign subfamilies to most of proteins.

We aimed to select the proteins with no orthologues in humans, however, in the target prioritization analysis, half of the selected proteins were clustered with human orthologues. Looking at the sequence identity of these proteins, we observed that all of them shared <60% of their identities. It has been shown that kinases with sequence similarities higher than 60% have very similar structures and the compounds that bind to their sites usually show similar structure-activity-relationships [13]. Thus, it is possible that the differences in the active sites of the orthologues can be explored and the binding of off-targets may be avoided. It also allows for the repurposing of approved drugs.

Our target prioritization approaches enabled us to find promising targets. Eight out of the 33 highly expressed kinases were prioritized as targets, meaning that our approaches were effective in selecting important targets. Many of those targets have been genetically validated in *T. brucei, e.g.*, Aurora kinase 1(AIRK) [46]; Casein kinase 1(CK1) [47]; Cyclin-dependent kinases CRK3 and CRK1 [48]; Glycogen synthase kinase 3(GSK3) [49]; WEE-1 like kinase [44]; and Polo-like kinase (PLK) [50]. Many of them are also being studied in *Leishmania* species. *L. major* and *L. donovani* AIRK have been cloned and characterized [51,52], their cellular locations elucidated during various phases of the *L. donovani* cell cycle [52], and chemical validation studies have shown similar effects compared to the *T. brucei* validation study [53,54]*.* CK1 has been identified as an hexokinase [55] capable of phosphorylating host proteins [56]. *L. major* CK1 has been targeted by human casein kinase inhibitors [57] and the kinase has also been chemically validated in *L. donovani* [58]*.* The cyclin-dependent kinases 2 – CRK1 and CRK3 were experimentally validated in *L. mexicana* as essential proteins [59] with fundamental roles during cell cycle progression [60]. Also, many inhibitor-driven studies have been carried in order to reach CRK3 inhibition, but few displayed *in vitro* activity [[61], [62], [63], [64]]. Recombinant GSK3 was expressed and purified in L. *major* and L. *infantum.* The crystal structure of *Lmaj*GSK3 was elucidated and eleven protein kinase inhibitors were tested against *Linf*GSK3, *Tbru*GSK3, and *Hsap*GSK3 enabling a structure-activity relationship comparison between the binding sites, which provides ways to predict inhibitors with binding modes that might be effective and selective [65]. Targeting leishmanial GSK3 led to cell cycle defects and apoptosis-like death [66]; a study with indirubin derivatives could enhance the selectivity of the inhibitors towards GSK3 over CRK3 [67]. *T. brucei* MKK1 and MKK5 knockout mutants were not essential for the parasite's survival or virulence [68]. In *L. mexicana*, MKK1 mutants have a shortened flagellum [69] and MKK5 activates MPK4, which has been proposed as a drug target [70].

Our drug repurposing approach led to the selection of 7 compounds with potential as *Leishmania* inhibitors. Exploration into the biological targets of parasites with orthologues in mammals has been avoided for decades, and it aims to eliminate possible problems of selectivity and adverse effects during the drug development process. However, the situation is radically different when this parasite protein is orthologous to a therapeutic drug target. In this case, orthologues can provide evidence of “druggability” [71]. Three out of seven compounds were active against L. *infantum,* L. *braziliensis* and *L. amazonensis* promastigotes. Trametinib was the most potent compound against L. *infantum* and L. *braziliensis* amastigotes. This drug reduced ~50% of intracellular amastigotes at 30 μM for L. *infantum* and at 15 μM for L. *braziliensis.* Trametinib is a drug used for the treatment of anaplastic thyroid cancer [72]. Its targets are mitogen-activated extracellular signal-regulated kinase 1 *(MEK1 or MAPKK1)*, *MEK2* activation, and MEK1 and *MEK2* kinase activity. MEK proteins are upstream regulators of the extracellular signal-related kinase (ERK) pathway, which promotes cellular proliferation [73]. The predicted trametinib targets in *Leishmania* are also MAPKK1 and MAPKK2. Both share 34% similarity with human MAPKK1. This reinforces the possibility of repurposing trametinib to a different application with similar mechanism of action. NMS-1286937 is an inhibitor of Polo-like kinase 1 (PLK-1), a key component of the cell cycle control machinery with important roles in the mitotic entry, centrosome duplication, bipolar mitotic spindle formation, transition from metaphase to anaphase, cytokinesis, and maintenance of genomic stability [74]. PLK-1 is also the predicted target of NMS-1286937 in *Leishmania*, with 48% of identity. This compound has good oral bioavailability in rodent and non-rodent species and has proven antitumor activity in different preclinical models against solid tumors and hematologic malignancies [75]. R1530 targets known cell cycle regulators like Aurora Kinase A and PLK4 [76]. R1530 inhibits tumor growth by blocking a variety of tumorigenic and angiogenic pathways. This compound did not present any observed toxicity at doses which resulted in significant growth inhibition, tumor regression, and a significant improvement in survival [77]. Although the EC_50_ of these compounds were not considered optimal against the Leishmania species tested (EC50 < 10 μM) [78], here we report only the first steps in a drug discovery pipeline. They might be used as scaffolds for compound optimization and hit selection. Further studies need to be conducted in order to establish *in vivo* efficacy of kinase inhibitors in experimental leishmaniasis model.

In conclusion, the bioinformatics pipeline used in this work allowed a very thorough classification of L. *infantum* and L. *braziliensis* kinomes – most of the kinases were assigned at subfamily level. The functions of these kinases were catalogued and compared to human and other *Leishmania* orthologues. Finally, we predicted 30 protein kinases that have the potential to be good drug targets. This information will be useful for the discovery of new leishmanicidal compounds. The drug repurposing pipeline allowed us to find a kinase inhibitor currently indicated for cancer treatments that has the potential to be repositioned for the treatment of leishmaniasis. The drug trametinib and the compounds NMS-1286937 and RG-1530 presented good to moderate inhibition of L. *infantum and L. braziliensis* amastigotes and might be used as scaffolds for future hit-to‑lead optimization.

The following are the supplementary data related to this article.

Supplementary Table S1: Leishmania infantum kinases classification following bioinformatics pipeline Supplementary Table 1

Supplementary Table S2: Leishmania braziliensis kinases classification following bioinformatics pipeline Supplementary Table 2

Supplementary Table S3: Ortholologous kinases between L. infantum, L. braziliensis, L. major; and blast comparisons with human kinases. Supplementary 3

Supplementary material Image 1

## Author contributions statement

Each author has contributed significantly to this work. JVBB, PIPR, ENM, NF, and CHA conceived and designed the experiments. JVBB, ACS, and PIPR performed the bioinformatics analyses and computational experiments. NG and DCM conducted the experimental assays. JVBB, ACS, PIPR, NG, DCM, ENM and CHA analyzed the data. JVBB, PIPR, ENM, and CHA wrote the paper. All authors read, edited, and approved the final manuscript.

## Declaration of Interests

None.
