# Supplementary material for: Unveiling the Kinomes of Leishmania infantum and L. braziliensis Empowers the Discovery of New Kinase Targets and Antileishmanial Compounds
Source: Comput Struct Biotechnol J. 2019 Feb 8;17:352–61. doi: 10.1016/j.csbj.2019.02.005 (PMC6429582; doi:10.1016/j.csbj.2019.02.005)
Supplement: Supplementary file 4 — Supplementary material [file mmc4.docx]

Supplementary Material

Unveiling the kinomes of *Leishmania infantum* and *L. braziliensis* empowers the discovery of new kinase targets and antileishmanial compounds

**Joyce Villa Verde Bastos Borba**^1^**, Arthur Carvalho Silva**^1^**, Pablo Ivan Pereira Ramos**^2^**, Nathalia Grazzia**^3^**, Danilo Ciccone Miguel**^3^**, Eugene N. Muratov**^4,5^**,** **Nicholas Furnham**^6^**, and Carolina Horta Andrade**^1^**^,*^**

^1^Labmol – Laboratory for Molecular Modeling and Drug Design, Faculdade de Farmácia, Universidade Federal de Goiás - UFG, Goiânia, GO, 74605-510, Brazil.

^2^Instituto Gonçalo Moniz (IGM), Fundação Oswaldo Cruz (FIOCRUZ), Salvador, BA, 40296-710, Brazil.

^3^LEBIL – Laboratory of studies of the Biology of Leishmania Infection, Department of Animal Biology, Biology Institute, State University of Campinas (UNICAMP), Campinas, SP, Brazil.

^4^Laboratory for Molecular Modeling, Division of Chemical Biology and Medicinal Chemistry, Eshelman School of Pharmacy, University of North Carolina, Chapel Hill, NC, 27599, USA.

^5^Department of Chemical Technology, Odessa National Polytechnic University, Odessa, 65000, Ukraine.

^6^Department of Pathogen Molecular Biology, London School of Hygiene and Tropical Medicine, London, UK.

[*carolina@ufg.br](mailto:*carolina@ufg.br)

**
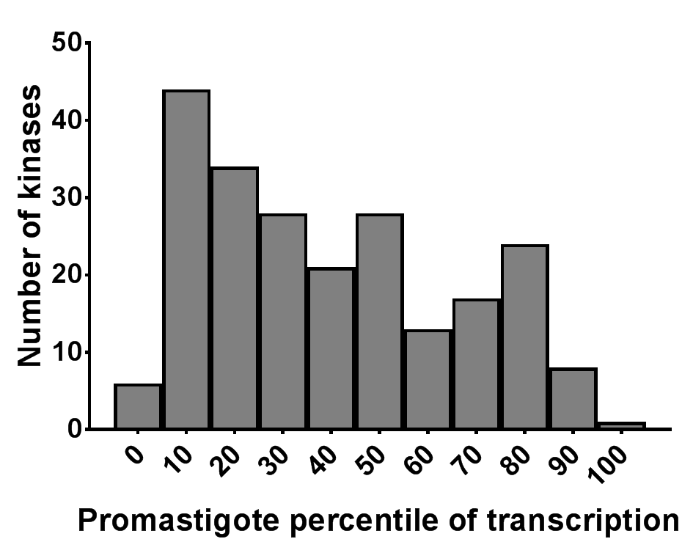
**

**Supplementary Figure S1. Transcript expression of *L. infantum* kinases.** The histogram shows the distribution of percentages of transcripts of protein kinases. The number of kinases in each expression interval is represented in the vertical axis.


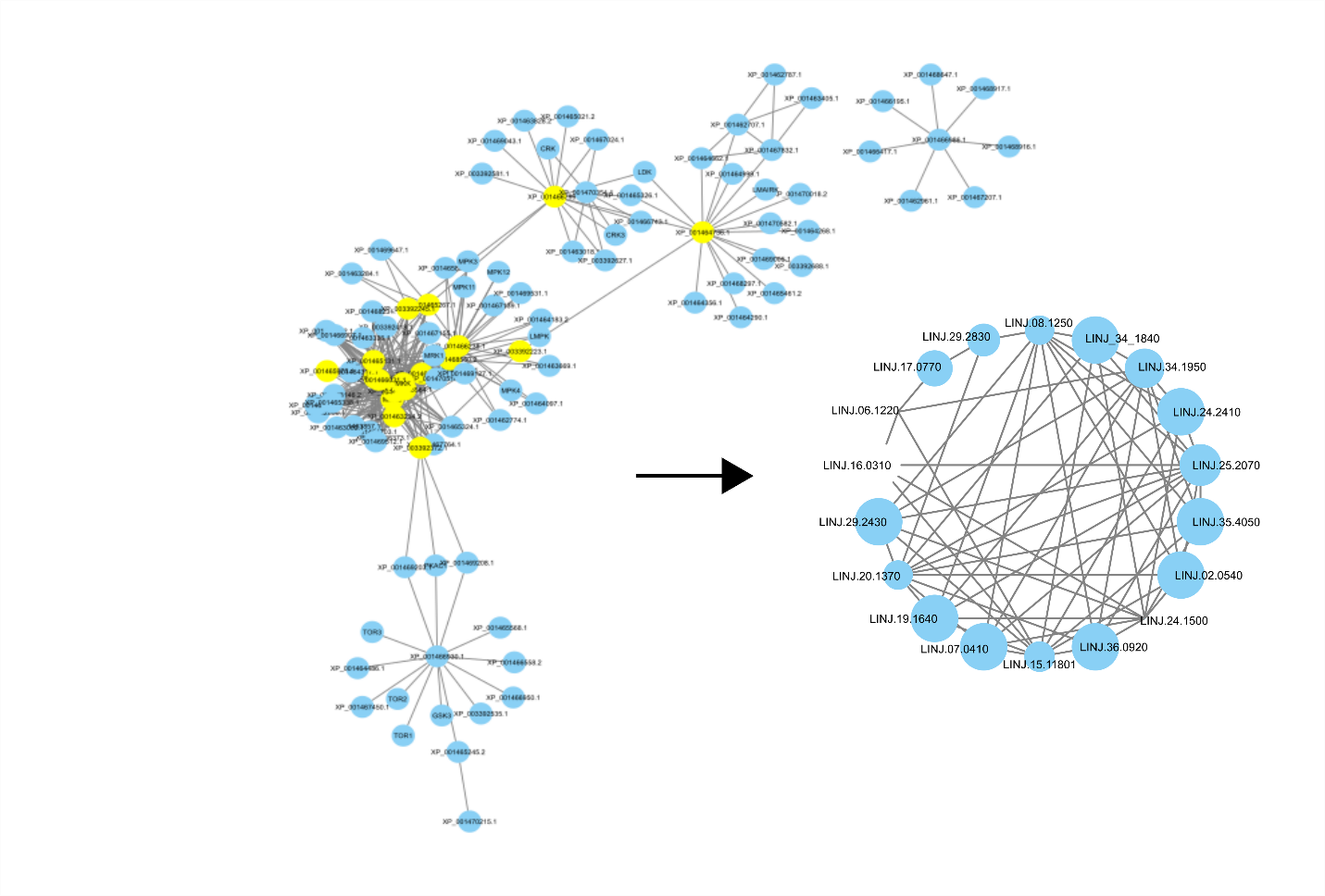


**Supplementary Figure S2. Target prioritization approach.** A protein-protein interaction network of kinome proteins was constructed using the web server STRING and a sub-network was extracted using CytONCA, a Cytoscape plugin that calculates graph centrality measures. According to this criterion, the most important nodes were output into that graph, leading to the target selection.

**
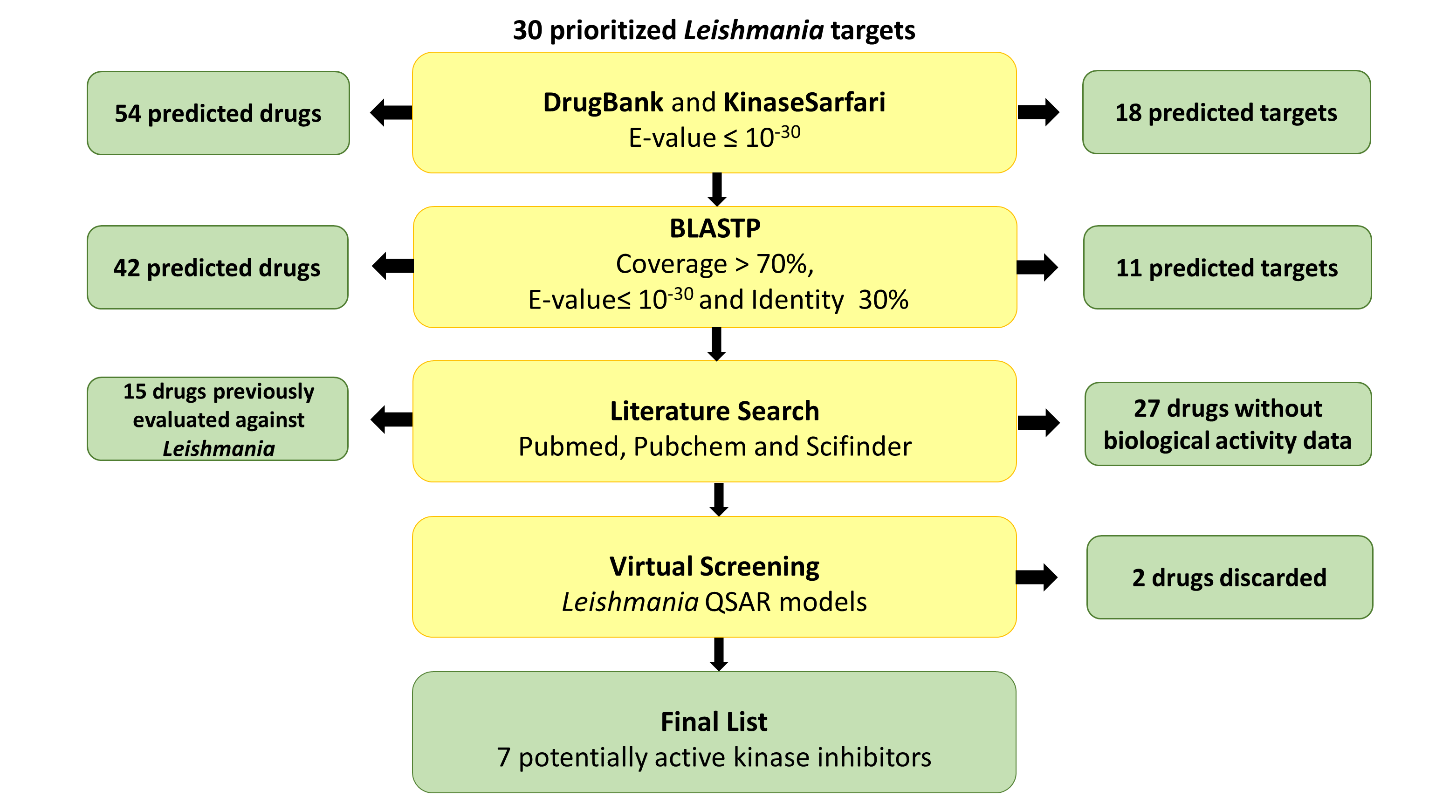
**

**Supplementary Figure S3. Flowchart summarizing the in silico repurposing strategy and corresponding results.** The green boxes represent the summarized results obtained at each stage of the study

**Supplementary Figure S4. Cytotoxicity of R1530, NMS-1286937 and Trametinib to BMDM cells.**

**Supplementary Table S4. Prioritized targets in essentiality and centrality measures approaches.**

| **ID** | **Classification** | **Functional Annotation** |
| --- | --- | --- |
| LinJ.05.0550 | CMGC/CDK/CRK2 | metabolism, signal transduction, cell growth and death, environmental adaptation |
| LinJ.16.1000 | CMGC/CDK/CRK4 | transcription, transport and catabolism, cell growth and death |
| LinJ.17.0770 | Other/PKL/PKL | signal transduction, cell growth and death |
| LinJ.18.0270 | CMGC/GSK | cell growth and death, signal transduction |
| LinJ.20.0310 | Atypical/PDHK | Metabolism |
| LinJ.20.0780 | STE/STE11/STE11-Unclassified |  |
| LinJ.20.1140 | PKL/PIKK/FRAP | environmental adaptation, metabolism, cell growth and death, signal transduction |
| LinJ.21.1320 | CMGC/CDK/CRK1 | signal transduction, cell growth and death |
| LinJ.27.2570 | CMGC/CDK/CRK6 | transcription, cell growth and death |
| LinJ.28.0550 | Other/AUR/AIRK | cell growth and death |
| LinJ.30.2930 | CMGC/MAPK/MAPK5 | Signal transduction, Cell motility, Cell growth and death, Cellular community, environmental adaptation |
| LinJ.35.1030 | CK1/CK1/CK1.2 | transcription, signal transduction |
| LinJ.36.0600 | CMGC/CDK/CRK3 | metabolism, cell growth and death, environmental adaptation |
| LinJ.02.0540 | STE/STE11 |  |
| LinJ.06.1220 | AGC/NDR |  |
| LinJ.07.0410 | STE/STE7 | environmental adaptation, cell growth and death, signal transduction |
| LinJ.08.1250 | STE/STE11/CDC15 |  |
| LinJ.16.0310 | STE/STE20 |  |
| LinJ.19.1640 | STE |  |
| LinJ.20.1370 | Other | signal transduction |
| LinJ.24.1500 | STE/STE11 | signal transdutction, environmental adaptation,metabolism |
| LinJ.24.2410 | STE/STE7-like/MKK4 | Signal transtuction, Cell motility, Cell growth and death, Cell community |
| LinJ.25.2070 | STE/STE11 | signal transduction, envionmental adaptation |
| LinJ.29.2430 | STE/STE7-like/MKK1 | environmental adaptation, cell growth and death, signal transduction |
| LinJ.29.2830 | Other/WEE | cell growth and death |
| LinJ.34.1840 | STE/STE7 | environmental adaptation,cell growth and death, signal transduction, cell motility, cell community |
| LinJ.34.1950 | Other | signal transduction |
| LinJ.35.4050 | STE | signal transduction |
| LinJ.36.0920 | STE/STE7 | environmental adaptation, cell growth and death, signal transduction, cell motility, cell community |

**Supplementary Table S5. Potential antileishmanial drugs and their potential targets revealed in this study**

| **ID** | **Classification** | **Associated Target** | **Coverage** | **E-value** | **Identity** | **Approved/ Clinical candidates** |
| --- | --- | --- | --- | --- | --- | --- |
| LinJ.05.0550 | CMGC/CDK/CRK2 | Cyclin-dependent kinase 2 | 96% | 8.00E-104 | 47% | Alvocidib, Dinaciclib, Seliciclib, RG-547, Milciclib, (7S)-Hydroxyl-Staurosporine, BMS-387032, Indirubin sulfate, TG-02, RGB-286638 |
| LinJ.16.1000 | CMGC/CDK/CRK4 | Cyclin-dependent kinase 5 | 97% | 2.00E-42 | 49% | Dinaciclib, Seliciclib, RGB-286638 |
| LinJ.17.0770 | Other/PKL/PKL | Serine/threonine-protein kinase PLK1 | 77% | 3.00E-76 | 48% | Volasertib, Bl2536, NMS-1286937, GSK-461364, TAK-960, MK-1496, Cafusertib, HMN-214 |
| LinJ.18.0270 | CMGC/GSK | Glycogen synthase kinase-3 beta | 73% | 1.00E-112 | 50% | LY-2090314, Tideglusib, Lithium |
| LinJ.21.1320 | CMGC/CDK/CRK1 | Cyclin-dependent kinase 5 | 97% | 2.00E-120 | 56% | Dinaciclib, Seliciclib, RGB-286638 |
| LinJ.27.2570 | CMGC/CDK/CRK6 | Cyclin-dependent kinase 2 | 95% | 4.00E-94 | 45% | Alvocidib, Dinaciclib, Seliciclib, RG-547, Milciclib, (7S)-Hydroxyl-Staurosporine, BMS-387032, Indirubin sulfate, TG-02, RGB-286638 |
| LinJ.28.0550 | Other/AUR/AIRK | Aurora Kinase A | 87% | 8.00E-83 | 44% | Alisertib, ENMD-981693, AT-9283, ENMD-2076, MLN-8054, KWW-2449, RG-1530, MK-5108, PF-0381435, TAS-119, CYC116 |
| LinJ.30.2930 | CMGC/MAPK/MAPK5 | Mitogen-activated protein kinase 1 | 93% | 3.00E-61 | 36% | Ulixartinib, GCD-0994, MK-8353 |
| LinJ.36.0600 | CMGC/CDK/CRK3 | Cyclin-dependent kinase 2 | 96% | 6.00E-29 | 58% | Alvocidib, Dinaciclib, Seliciclib, RG-547, Milciclib, (7S)-Hydroxyl-Staurosporine, BMS-387032, Indirubin sulfate, TG-02, RGB-286638 |
| LinJ.29.2430 | STE/STE7-like/MKK1 | Dual specificity mitogen-activated protein kinase kinase 1 | 75% | 2.00E-48 | 34% | Trametinib dimethyl sulfoxide, cobimetinib, cobimetinib fumarete, selumetinib, binimetinib, Cl-1040, PD-0325901, Pimasertib, Refametinib |
| LinJ.36.0920 | STE/STE7/MKK5 | Dual specificity mitogen-activated protein kinase kinase 1 | 84% | 1.00E-54 | 34% | Trametinib dimethyl sulfoxide, cobimetinib, cobimetinib fumarete, selumetinib, binimetinib, Cl-1040, PD-0325901, Pimasertib, Refametinib |
